# Transcriptional heterogeneity in the lactase gene within cell-type is linked to the epigenome

**DOI:** 10.1038/srep41843

**Published:** 2017-01-31

**Authors:** Edward Oh, Richie Jeremian, Gabriel Oh, Daniel Groot, Miki Susic, KwangHo Lee, Kelly Foy, Peter W. Laird, Arturas Petronis, Viviane Labrie

**Affiliations:** 1 Krembil Family Epigenetics Laboratory, Centre for Addiction and Mental Health, Toronto, Ontario, Canada; 2 Center for Epigenetics, Van Andel Research Institute, Grand Rapids, MI, USA; 3 Center for Neurodegenerative Science, Van Andel Research Institute, Grand Rapids, MI, USA

## Abstract

Transcriptional variation in histologically- and genetically- identical cells is a widespread phenomenon in tissues, yet the processes conferring this heterogeneity are not well understood. To identify contributing factors, we analyzed epigenetic profiles associated with the *in vivo* transcriptional gradient of the mouse lactase gene (*Lct*), which occurs in enterocytes along the proximal-to-distal axis of the small intestine. We found that epigenetic signatures at enhancer and promoter elements aligns with transcriptional variation of *Lct* in enterocytes. Age and phenotype-specific environmental cues (lactose exposure after weaning) induced changes to epigenetic modifications and CTCF binding at select regulatory elements, which corresponded to the alterations in the intestinal *Lct* mRNA gradient. Thus, epigenetic modifications in combination with CTCF binding at regulatory elements account for the transcriptional gradient in *Lct* in cells of the same type. Epigenetic divergence within enterocytes may contribute to the functional specialization of intestinal subregions.

Within a tissue, seemingly identical cells have been found to exhibit a large degree of variation in their transcriptomes^1^,^2^. Although this cell-to-cell variation can be partially stochastic^3^, there are coordinated transcriptional gradients in cells of the same type across whole organs. For example, prominent transcriptional gradients have been detected in intestinal epithelial cells (enterocytes) along the length of the small intestine^4^,^5^,^6^, in hepatocytes across liver zones^7^,^8^,^9^, in myocytes in different heart chambers^10^−^13^, and in adipocytes located in bone marrow^14^. Gradients in transcriptional states enable cells that are histologically identical to perform biologically distinct roles, vary in their response to environmental stimuli, and exhibit differential disease vulnerability^1^,^15^. Despite its evident biological significance, the molecular processes coordinating same cell-type transcriptional divergence across tissue subregions are not well understood.

Epigenetic mechanisms could contribute to transcriptional variation within the same cell type, as epigenetic modifications are important in regulating gene transcription^16^, determining cell identity^17^,^18^, and affecting genomic functions in response to aging and environmental cues^19^. Recently, DNA modification analysis of single cells from *in vitro* cultures found significant heterogeneity^20^,^21^,^22^, suggesting the potential for epigenetic marks to differ between cells of the same type in living organisms. In this study, we sought to examine the divergence of DNA modifications within a cell type *in vivo* and explored its potential in regulating same cell type transcriptional gradients in tissue. To answer this question, we examined transcriptional and epigenetic variation of the lactase gene (*Lct*) in enterocytes along the proximal-to-distal axis of the mouse small intestine. *Lct*, which is responsible for lactose metabolism, represents an ideal model to identify DNA modifications that can contribute to transcriptional heterogeneity for tissue subregion specialization, as it exhibits a distinguished transcriptional gradient in enterocytes; a gradual elevation from duodenum to jejunum, followed by a steady decline to the ileum^23^,^24^,^25^. Recent studies have shown that *Lct* mRNA expression is dependent upon DNA modifications at key genomic regulatory elements^18^,^26^, however, the extent to which DNA modifications diverge to regulate *Lct* transcription along the length of the intestine remains uninvestigated. In addition to this, we also examined how aging and the environment can modify *Lct* expression through DNA modifications at *Lct* genomic regulatory elements.

Here, we found that in enterocytes isolated from different intestinal regions, DNA modifications at specific genomic regulatory elements were concordant with the transcriptional variation of *Lct*. Aging and environmental exposures (lactose feeding after weaning) resulted in DNA modification changes at these regulatory sites and changes in *Lct* transcriptional patterns. Overall, DNA modification patterns support the aging- and environmentally-induced gradients of *Lct mRNA* and, more broadly, could affect phenotypic outcome by modifying transcriptional programs within same cell types.

## Results

### Within cell-type differences in *Lct* mRNA are correlated to epigenetic alterations

**Figure 1:**
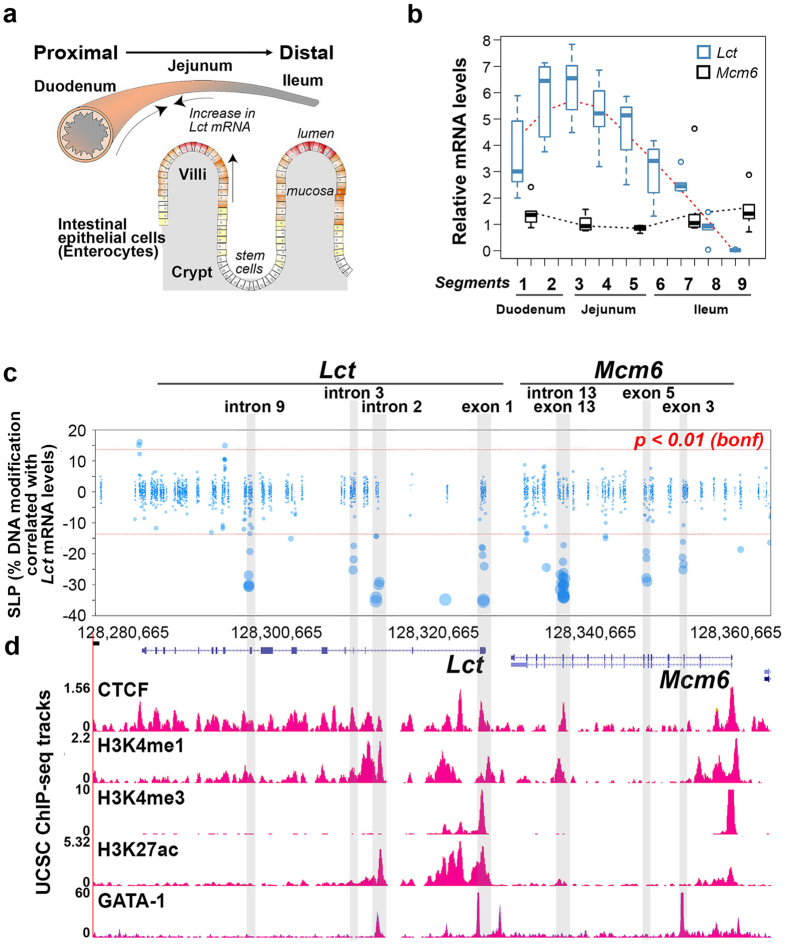
Within cell-type variation in *Lct* mRNA along the proximal-to-distal axis of small intestine is correlated to DNA modification differences at genomic regulatory elements. (**a**) Illustration of the proximal-to-distal axis of the small intestine and villi. (**b**) There is a segment-specific gradient in steady-state *Lct* mRNA levels. There is no transcriptional gradient in *Mcm6* along the intestine. Villin-1 was used as an endogenous control. Data are represented as mean ± SEM. n = 5–7 mice. **p < 0.01, ***p < 10^−3^ by Tukey’s HSD post hoc test in comparison to segment 3. (**c**) Enterocyte-specific DNA modification clusters (>3 cytosine modifications) within the *Lct*–*Mcm6* locus significantly correlated with *Lct* mRNA levels (n = 53; 9 segments × 5–6 mice per segment). Bonferroni correction for multiple testing (*p* < 0.01) is indicated by dash lines. SLP refers to the -log *p*-value of the Pearson’s correlation R^2^ (negative values signify an inverse correlation). (**d**) DNA modification clusters associated with the *Lct* transcriptional gradient (highlighted by grey vertical bars) overlap histone marks characteristic of promoters and enhancers, as well as the transcription factor GATA1 and the chromatin architectural protein CTCF (ChIP-seq data from ENCODE, n = 2).

Enterocytes exhibit differing *Lct* transcriptional patterns along the proximal-distal axis of the small intestine, which enables intestinal subregion specialization in lactose metabolism (Fig. 1a). We examined *Lct* mRNA levels in enterocytes from nine segments of the adult (postnatal day 60; P60) mouse small intestine; the proximal, middle and distal segments of the duodenum (segment 1–2), jejunum (segment 3–5), and ileum (segment 6–9), and observed a gradient in steady-state *Lct* mRNA levels (main effect of segment: *F*_8,46_ = 23.4*, p* = 8.4 × 10^−14^; Fig. 1b). *Lct* mRNA levels were highest in the proximal jejunum (segment 3), and then gradually declined toward the proximal duodenum (*p* = 0.008 compared to segment 3) and distal ileum (*p* = 1.2 × 10^−10^). In contrast, *Mcm6* mRNA levels did not change across the intestinal segments. This indicates that enterocytes from the mid-duodenum to mid-jejunum are most specialized for the digestion of lactose.

**Figure 2:**
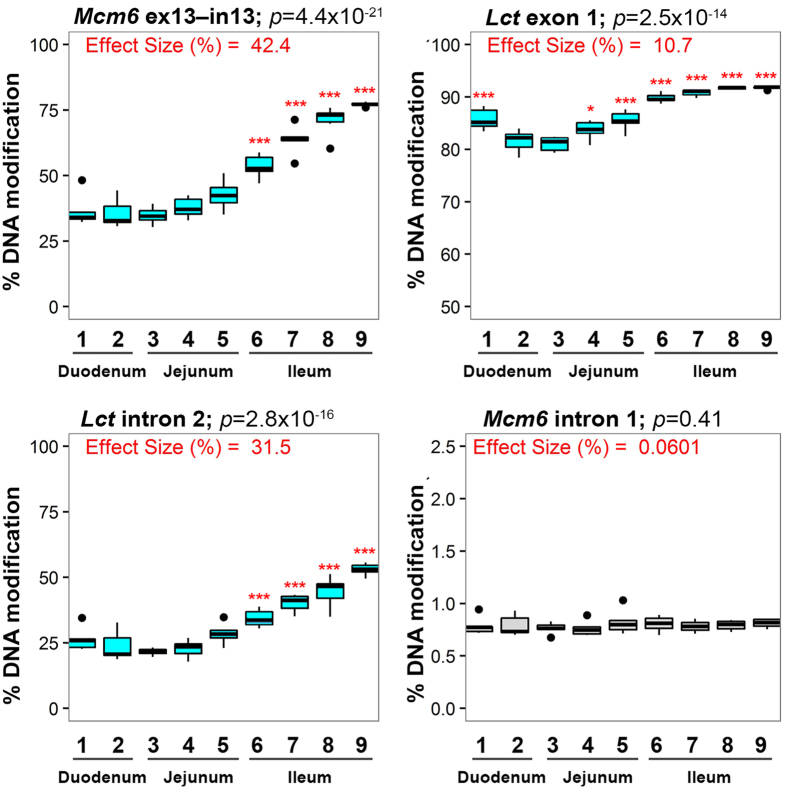
Divergence of DNA modification at *Lct* regulatory elements in enterocytes across intestinal segments. Average % DNA modification (y-axis) at *Mcm6* exon 13-intron 13, *Lct* intron 2 and *Lct* exon 1 is significantly associated with *Lct* transcriptional variation along the proximal-to-distal axis of the intestine (x-axis). *Mcm6* exon 13-intron 13, *Lct* intron 2 and *Lct* exon 1 regions selected based on findings of Fig. 1c. Average % DNA modification of *Mcm6* intron 1 included as a negative control. *P*-value obtained by one-way ANOVA (n = 53; 9 segments × 5–6 mice per segment). *p < 0.05, ***p < 10^−3^ by Tukey’s HSD post hoc test in comparison to segment 3.

Using a targeted bisulfite sequencing approach^27^, we sought to determine whether DNA modifications (methylation and other cytosine modifications) could account for this within cell-type transcriptional gradient. In enterocytes, selectively isolated from the villi of each of the nine intestinal segments, we characterized 7,580 cytosines (609 CpG sites & 6,971 CpH sites) along the *Lct* and its neighbouring gene, *Mcm6.* We included *Mcm6* in our investigation because DNA variation in this gene, particularly at *MCM6* intron 13, affects inter-individual differences in lactase levels in humans^28^,^29^,^30^. We identified several clusters of modified cytosines (3 or more in <500 bp) which showed significant inverse correlations with *Lct* mRNA levels (R^2^ = 0.62–0.85; *p* < 0.01 after Bonferroni correction; Fig. 1c). Most notably, at *Mcm6* exon 13–intron 13, we observed a large cluster modified cytosines associated with *Lct* mRNA variation across the intestine (18 CpGs and 1 CpH site, *p* = 3.3 × 10^−3^ to 1.4 × 10^−11^ after Bonferroni correction; R^2^ = 0.67–0.85). DNA modification densities in this region were lowest in segment 3 (34.7%) and reached maximal levels in the distal ileum (77.1%; *p* = 4.4 × 10^−21^; Fig. 2). There were also DNA modification clusters strongly correlated with *Lct* mRNA at *Lct* exon 1 and *Lct* intron 2 (each genomic site containing 6 CpGs, R^2^ = 0.62–0.85; *p* = 1.8 × 10^−2^ to 4.0 × 10^−12^ after Bonferroni correction; Figs 1c and 2). Thus, DNA modifications at specific regions are related to the *Lct* transcriptional gradient within enterocytes along the small intestine.

We next examined ENCODE data of adult mouse small intestine^31^ to determine whether the sites we associated with transcriptional variation in *Lct* could function as genomic regulatory elements (Fig. 1d). We found histone marks indicative of a poised element at *Mcm6* exon 13–intron 13 (H3Kme1), an active enhancer at *Lct* intron 2 (H3K4me1 & H3K27ac), and an active promoter at *Lct* exon 1 (H3K4me3 & H3K27ac). *Mcm6* exon 13–intron 13, *Lct* exon 1 and intron 2 showed binding of CTCF, a protein that facilitates interactions between transcription regulatory sequences by affecting chromatin architecture^32^,^33^. In addition, *Lct* exon 1 and intron 2 overlapped GATA1 binding sites. GATA1 is a member of GATA transcription factor family that modulates *Lct* expression^34^, and modifies epigenetic marks to promote subregion transcriptomic differences in the small intestine^35^. Thus, sites in which DNA modifications were significantly predictive of regional differences in *Lct* contained chromatin signatures of enhancers and a promoter, along with an enrichment in transcriptional regulatory proteins.

### The *Lct* transcriptional and epigenetic gradients in enterocytes are age-dependent

**Figure 3:**
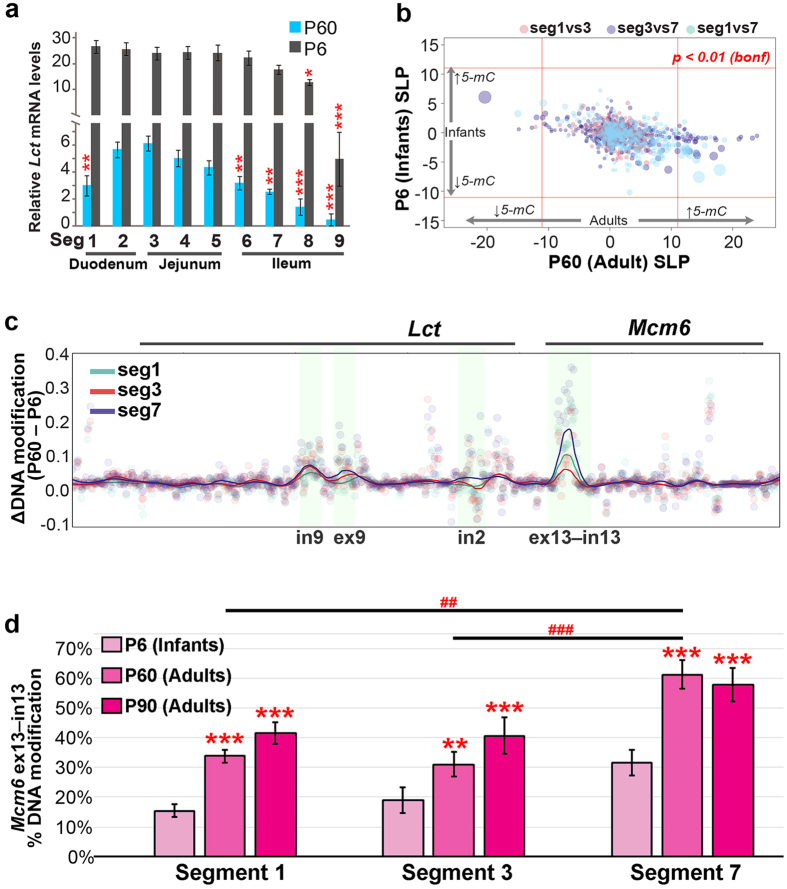
Regional differences in *Lct* transcription arises with age and the divergence of DNA modification patterns within enterocytes. (**a**) Infant mice (P6; n = 6) had higher *Lct* mRNA levels than adults (P60; n = 4–6), and had an attenuated transcriptional gradient. Data are represented as mean ± SEM. *p < 0.05, **p < 0.01, ***p < 10^−3^ by Tukey’s HSD post hoc test compared to age-matched segment 3. (**b**) Comparison of individual DNA modifications between intestinal segments (within age group) revealed a significant divergence in DNA modifications in villi enterocytes along the intestine of adults (x-axis), but not infants (y-axis). SLP refers to signed log *p*-value. Significant comparisons exceed the dashed red line (*p* < 0.01 after Bonferroni correction). (**c**) Identification of genomic sites exhibiting differential DNA modification with age. The *Mcm6* exon 13–intron 13 site in segment 7 (blue) displayed the largest increase in % DNA modification with age, with segment 1 (red) and segment 3 (turquoise) showing smaller DNA modification increases. There was a smaller gain in DNA modifications at *Lct* exon 9–intron 9 in adults. Modification density differences y-axis; average % DNA modification in adults minus in infants by individual CpGs (n = 632) fitted with LOESS curve for each segment. (**d**) Average % DNA modification densities at *Mcm6* exon 13–intron 13 site (Data are represented as mean ± SEM; n = 14 CpGs) in enterocytes along the intestine of infants at P6 and adults at P60 and P90. DNA modification levels increased with age at *Mcm6* exon 13–intron 13, particularly in segment 7 enterocytes. Data are represented as mean ± SEM. **p < 0.01, ***p < 10^−3^ by Tukey’s HSD post hoc test compared to same segment of infant mice. ^##^*p* < 0.01, ^###^*p* < 10^−3^ by Tukey’s HSD post hoc test for between segments of P60 adults. (**b**–**d**) n = 3–7 per age group for each segment.

We investigated whether transcriptional gradients could be established from age-associated epigenetic changes. To do this, we first examined *Lct* mRNA levels in enterocytes across the small intestine of infant mice (P6) and adult mice (P60). *Lct* mRNA levels in infants were mostly similar across intestinal segments, and were 5–15 fold higher than adults (main effect of age: *F*_8,85_ = 13.23*, p* = 3.12 × 10^−12^; Fig. 3a). This signifies that transcriptional differences in *Lct* along the small intestine manifests with age, where over time *Lct* becomes mainly suppressed in enterocytes of the proximal duodenum and ileum relative to medial segments.

Next, the extent to which DNA modifications diverge with age at the *Lct-Mcm6* locus was examined in villi enterocytes from different intestinal regions. In adults, segments 1, 3 and 7 (duodenal, jejunal and ileal segments) differed substantially in *Lct* abundance, and consistently, DNA modifications differed significantly between adult segments (*p* < 0.01 after Bonferroni correction; Fig. 3b, x-axis). In infants, no significant DNA modification differences were observed between the intestinal segments (Fig. 3b, y-axis), which reflects their lack of an *Lct* transcriptional gradient.

We then searched for epigenetically-controlled DNA sites that could contribute to the age-dependent establishment of the *Lct* transcriptional gradient. For this, we investigated DNA modifications at the *Lct*–*Mcm6* locus in isolated villi enterocytes of adult and infant mice, comparing only matched segments between the age groups (Fig. 3c). We found differences in CpG (but not CpH) modifications with age in enterocytes along the intestine. In particular, *Mcm6* exon 13–intron 13 exhibited the highest localized gain of DNA modifications in adulthood (Fig. 3c). The age-related DNA modification increase at *Mcm6* exon 13–intron 13 was most prevalent in the segment with the largest *Lct* mRNA loss (segment 7; age-segment interaction: *F*_7,78_ = 5.78*, p* = 4.0 × 10^−4^; Fig. 3d). DNA modification densities in *Mcm6* exon 13–intron 13 increased by 30% in segment 7 in P60 adults compared to infants (*p* = 1.3 × 10^−4^), while there was an 18% increase segment 1 (*p* = 1.3 × 10^−4^) and a 12% increase in segment 3 (*p* = 0.0013; Fig. 3d). In older adults (P90), DNA modification levels continued to rise (by ~10%) in segment 1 and 3 (*p* = 0.025; Fig. 3d), indicating continued epigenetic aging at this *Mcm6* site. Thus, in addition to enabling lactase persistence in certain human populations^28^,^29^, epigenetic regulation of *Mcm6* exon 13–intron 13 could be central to the age-dependent decline of *Lct* in proximal and distal portions of the small intestine, leading to their inability to metabolize lactose. Thus, cells of the same type can exhibit a divergence in DNA modifications with age across tissue regions, which in turn may facilitate transcriptional gradients and the functional specialization of tissue subregions.

### The environment modifies the *Lct* transcriptional gradient and induces epigenetic alterations

**Figure 4:**
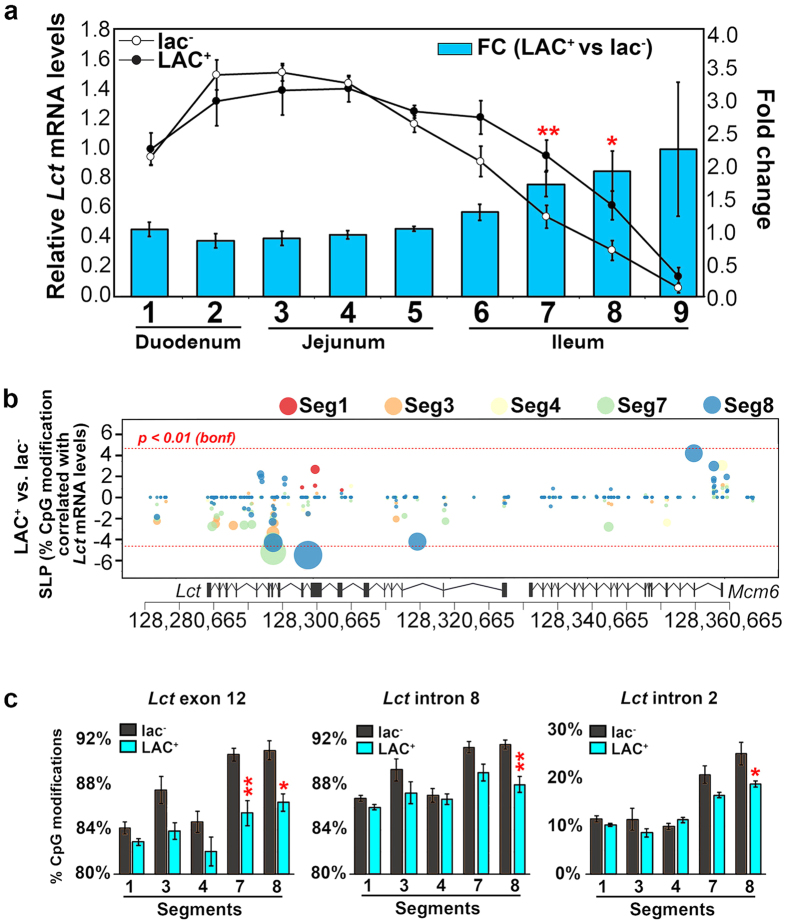
Lactose-treatment induces DNA modification changes in intestinal subregions that correspond to changes in the *Lct* transcriptional gradient. (**a**) Mice given lactose-containing milk (LAC^+^, n = 6) for 60-days had segment-specific increases in *Lct* mRNA in comparison to mice given lactose-free milk (lac^−^, n = 7). *Lct* mRNA following lactose exposure was increased in the ileum (seg7–8). *p < 0.05, **p < 0.01 by one-way ANOVA. (**b**) Changes in CpG modification were detected in intestinal segments that displayed *Lct* mRNA changes in response to lactose treatment (*p* < 0.01 after Bonferroni correction; dashed red line; LAC^+^ n = 6–7 mice per segment; lac^−^ n = 5–7 mice per segment). (**c**) Reductions in CpG modifications occurred in response to lactose treatment at *Lct* exon 12 (Factorial ANOVA; *F*_4,55_ = 2.3, *p* = 0.074), intron 8 (*F*_4,55_ = 2.3, *p* = 0.074) and intron 2 (*F*_4,55_ = 3.1, *p* = 0.023) in distal intestinal segments. Data are represented as average % CpG modification density ± SEM. Effect of lactose treatment by intestinal segment was calculated by factorial ANOVA. *p < 0.05 **p < 0.01, and ***p < 0.01 by Tukey’s HSD post hoc test for between lactose treatment within intestinal segment.

We determined whether a phenotype-related environmental signal (i.e. lactose exposure) could modify transcriptional patterns and induce epigenetic changes in enterocytes along the small intestine. For this experiment, we fed mice either lactose-containing milk (LAC^+^) or lactose-free milk (lac^−^) for 60 days. In response to lactose treatment, *Lct* mRNA levels increased in the enterocytes of distal intestinal segments (segment-diet interaction *F*_8,88_ = 5.2*, p* = 2.0 × 10^−5^; Fig. 4a). Specifically, lactose exposure increased *Lct* mRNA levels in enterocytes of segments 7 (*p* = 0.007) and 8 by 1.3- to 2- fold (*p* = 0.023) compared to mice in the lactose-free group (Fig. 4a).

To test whether the epigenome could be involved in the environmentally-induced changes in *Lct* mRNA across the intestine, we investigated DNA modifications in isolated villi enterocytes at the *Lct*–*Mcm6* locus in LAC^+^ and lac^−^ treated mice. We evaluated intestinal segments in which *Lct* was induced (segments 7 and 8) or showed no statistically significant change (segments 1, 3 and 4). Distal intestinal segments exhibited a significant decrease in CpG modification densities at *Lct* exon 12 (segment 7 = 5.2%, *p* = 4.7 × 10^−4^; segment 8 = 4.7%, *p* = 0.010), *Lct* intron 8 (segment 8 = 3.6%, *p* = 3.6 × 10^−3^), and *Lct* intron 2 (segment 8 = 6.4%; *p* = 0.012; Fig. 4b,c). CpG modifications were not changed significantly in segments 1, 3 and 4 (Fig. 4b,c). Although epigenetic modifications at *Mcm6* exon 13–intron 13 were found to be important for aging and regional differences in *Lct*, this genomic site was unchanged by milk-feeding, which could partly explain why *Lct* mRNA levels were not returned to that of pre-weaned infants after prolonged lactose exposure.

### CTCF binding along the intestine targets epigenetically-controlled sites associated with *Lct* transcriptional variation

**Figure 5:**
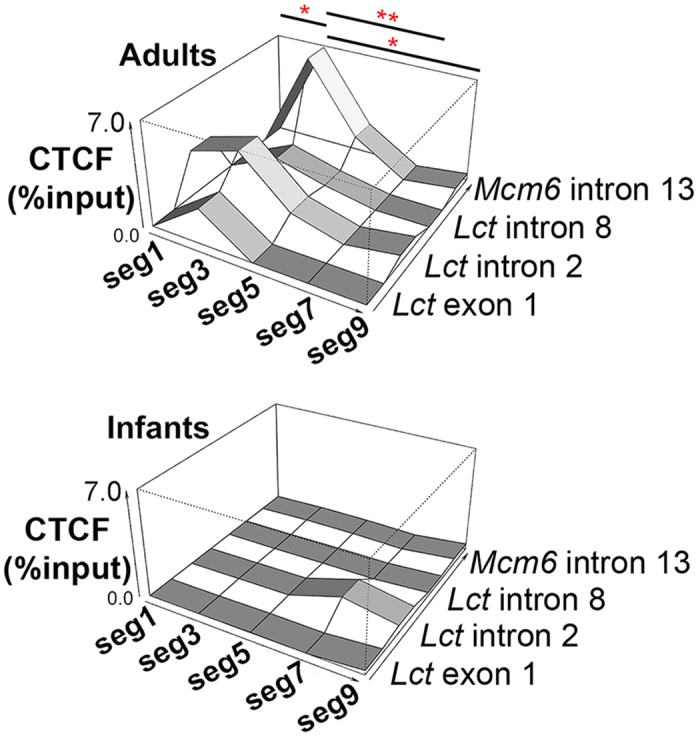
CTCF binding at epigenetically-controlled sites in the *Lct*–*Mcm6* locus corresponds to the *Lct* transcriptional gradient. ChIP-qPCR analysis of CTCF binding at *Mcm6* intron 13, *Lct* intron 8, *Lct* intron 2 and *Lct* exon 1 in adults (P60; top) and infants (P6; bottom) by segment (Data are represented as mean; n = 3 per group). Data presented as percent input after background normalization. *p < 0.05 and **p < 0.01 by Tukey’s HSD post hoc test for one-way ANOVA by effects of intestinal segment.

We wanted to examine the potential mechanism behind how DNA modification status at the *Mcm6* exon 13–intron 13 region located ~13 kb upstream of the *Lct* gene was able to exert such strong effects on the *Lct* promoter. We chose to examine the contribution of the chromatin architectural protein CTCF because of its ability to mediate architectural DNA interactions that facilitate transcription^36^. Furthermore, sequence-dependent CTCF occupancy can be regulated by DNA methylation status^37^,^38^,^39^,^40^,^41^ which leads to differential splicing and gene transcription. The contribution of CTCF binding in the *Lct-Mcm6* locus in mediating transcriptional gradients in cells of the same type across the adult and infant mouse small intestine was investigated by ChIP-qPCR (Fig. 5). In adults, CTCF binding was highest in the proximal jejunum (segment 3) and gradually declined in more proximal and distal intestinal regions (main effect segment: *F*_*4,19*_ = 5.12, *p* = 0.0083). Segment differences in CTCF binding were most apparent at *Mcm6* exon 13–intron 13, and to a lesser extent at *Lct* intron 2. In infant mice, on the other hand, we found that CTCF was absent from *Lct*–*Mcm6* regulatory elements (Fig. 5).

## Discussion

**Figure 6:**
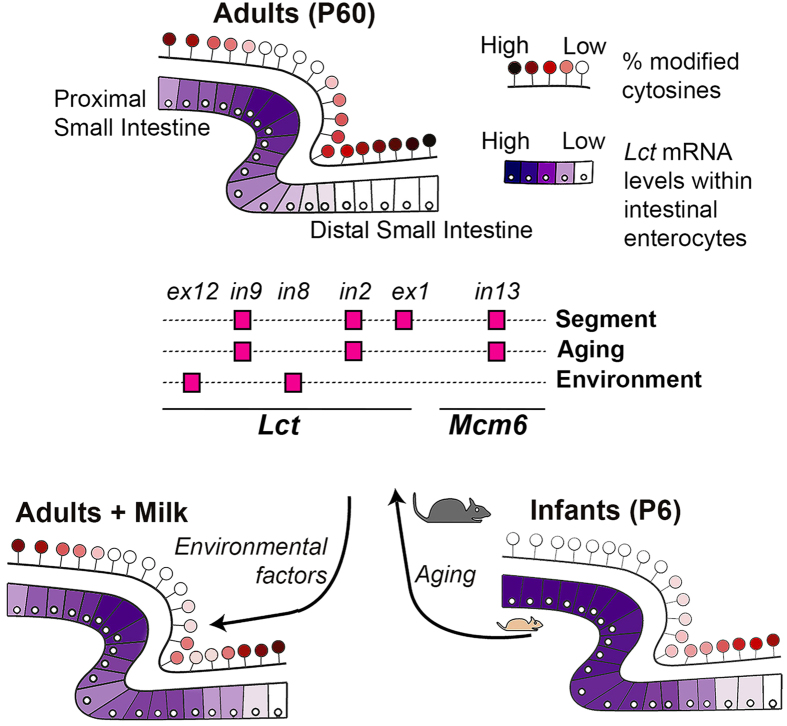
Relationship between *Lct* transcriptional gradient and DNA modification status. In enterocytes along the small intestine, transcriptional variation in the *Lct* gene corresponds to variation in DNA modification profiles at regulatory elements. The *Lct* transcriptional gradient is established with age and is modifiable by environmental signals; changes which co-occur with DNA modification density alterations in enterocytes at the same intestinal regions. The key genomic sites exhibiting DNA modification differences predictive of *Lct* transcriptional heterogeneity across intestinal segments and in response to aging and environmental signals are summarized.

Our results indicate that divergent epigenetic programming enables aging- and environmentally-induced changes in gene transcription occurring in cells of the same type (Fig. 6). In particular, the *Mcm6* exon 13–intron 13 site was found to be a key modulator of the age-dependent establishment and maintenance of the *Lct* transcriptional gradient. These findings are consistent with reports that this upstream region is an enhancer of *Lct* transcription based on genetic (lactase persistence SNPs in various populations)^28^,^30^ and molecular evidence that demonstrate its ability to regulate *Lct* transcription *in vitro*^42^,^43^,^44^. Our new observation that epigenetic control of *Mcm6* exon 13–intron 13 is important to *Lct* regulation in both mice and humans^26^,^28^,^29^ adds an epigenetic layer to these previous findings. Our study also suggests an evolutionary conservation of epigenetic regulation, which appears to precede genetic polymorphisms for the evolutionarily favorable lactase persistence trait^45^,^46^. Furthermore, the *Mcm6* exon 13–intron 13 site was not sensitive to environmental cues (i.e. milk), indicative that age-dependent epigenetic programming of *Mcm6* exon 13–intron 13 is not malleable. By contrast, other regulatory elements affecting the *Lct* gradient (i.e. *Lct* intron 2 and intron 8) remained epigenetically adaptive to environmental signals, enabling a partial recovery of *Lct* expression in the adult intestine after weaning. Therefore, localized DNA modification changes accumulating with age may facilitate the intestinal *Lct* transcriptional gradient, while some remain partially dynamic to environmental signals.

CTCF occupancy was concordant with the *Lct* transcriptional gradient along the small intestine, and inversely correlated to DNA modification profiles at these genomic regulatory elements, particularly at *Mcm6* exon 13–intron 13. CTCF is a multifunctional protein that participates in many epigenetic regulatory functions, including insulation via enhancer blocking, imprinting, X chromosome inactivation, and both transcriptional activation and repression^47^,^48^. CTCF can also influence DNA modification distribution both locally, through binding to chromatin boundaries, and distally, through effects on chromatin architecture^37^,^49^,^50^. Here, our findings indicate that CTCF binding at the *Lct*–*Mcm6* locus functions as an intestinal region-specific transcriptional activator in adult mice, potentially by enabling DNA looping of distal enhancers, such as the *Mcm6* exon 13–intron 13 locus^32^. CTCF binding may limit the accumulation of cell-type specific DNA modifications with age at middle regions of the intestine relative to distal regions during development, whereas the absence of CTCF could promote downregulation of *Lct* through unobstructed accumulation of DNA modifications and epigenetic silencing. On the other hand, the lack of CTCF at *Lct*–*Mcm6* sites in infant mice signifies that CTCF binding is not required for the expression of high *Lct* mRNA per se. Rather, CTCF occupancy (and its putative effects on chromatin structure) occurs in tandem with DNA modification changes following weaning, to prevent uniform epigenetic silencing of *Lct* with age. The resulting effect in adulthood is that CTCF and the opposing DNA modification landscape work in concert to facilitate a transcriptional gradient in *Lct* across the intestine.

DNA modifications and CTCF may work in tandem with transcription factors to create and maintain age-dependent transcriptional gradients in cells of the same type. Transcription factors can interact with gene enhancers, including those affecting *Lct*^25^,^44^. Transcription factors have also been shown to play a role in enterocyte differentiation along the crypt-to-villi axis (i.e., HNF-1a) and in intestinal subregion specialization (i.e., GATA4)^35^,^51^,^52^,^53^. For example, GATA4 has been shown to be upregulated in the proximal small intestine and downregulated in the distal small intestine to enable the development and divergence of duodenal and ileal portions of the intestine^5^,^6^. Together, our findings suggest an interplay between molecular and epigenetic factors that facilitate the biological specialization of cell of the same type. The molecular mechanism behind this concerted effort of CTCF, DNA modifications, and transcription factors at enhancer region(s) should be investigated in future studies.

Profiling epigenetic modifications of individual cell types in a tissue region-specific manner could offer insights into tissue specialization. Our findings emphasize the future studies should examine epigenetic contributions to the transcriptional divergence of numerous genes within cells of the same type, as these could help explain why tissue subregions can perform diverse biological functions^4^,^10^,^14^ and vary widely in disease susceptibility and treatment^54^,^55^. In addition, our findings provide an important lesson for epigenetic studies of phenotypes, as failure to consider within cell-type transcriptional variation and epigenetic divergence limits the detection of biologically significant effects. Future epigenetic and gene regulation studies in health and disease will be greatly refined by not only isolating the cell type of interest, but by sampling a single cell type across tissue subregions, and across aging and environmental parameters.

## Methods

### Mouse intestinal samples and milk treatments

Infant C57BL/6NCrl mice at postnatal day 6 (P6) and adult mice at postnatal day 60 (P60) and 90 (P90) were used to investigate epigenetic changes across the small intestine (segments 1–9). To investigate diet/environmental associated epigenetic changes, P30 mice were supplied *ad libitum* sterile food and either 2% lactose-containing milk or lactose-free milk in the place of water for 60 days. All animal procedures were approved by the Institutional Animal Care Committee of the Toronto Centre for Phenogenomics (TCP) and compiled per the requirements of the Canadian Council on Animal Care and Province of Ontario Animals for Research Act.

### RNA extraction and quantitative PCR

Enterocyte *Lct* mRNA levels were examined in each of the nine intestinal segments. For each segment, a small proximal portion (~30 mg) was homogenized with a ceramic bead-based homogenizer. Total RNA was extracted using Qiagen RNeasy Mini Kit with Qiagen RNase-free DNase I. RNA yield was quantified using NanoDrop ND-1000 (Thermo Fisher Scientific), and RNA integrity was verified via the Agilent Bioanalyzer 2100 system (Agilent Technologies). Purified RNA was converted to cDNA using High Capacity RNA-to-cDNA Kit (Life Technologies). *Lct* (Mm01285112_m1) and *Mcm6* (Mm00484848_m1) mRNA levels were quantified using TaqMan Gene Expression Master Mix (Life Technologies) using Applied Biosystems ViiA 7 real-time PCR system. The enterocyte marker *Villin-1* (Mm00494146_m1) mRNA was used as endogenous control for both *Lct* and *Mcm6* gene expression. ΔΔCt was used to calculate the relative steady-state mRNA levels of each sample. Analysis was performed using repeated-measures (RM) ANOVA, and significant interactions were analyzed by Tukey’s honest significant difference (HSD) post hoc comparisons. *Mcm6* mRNA levels did not display tissue subregion variations, indicating that within cell-type transcriptional gradients can be gene specific.

### Fine-mapping of DNA modifications in enterocytes

Enterocytes were isolated from only the villi of the small intestine segments (see Supplementary methods). The purity and specificity of the villi enterocyte isolation was confirmed to be 84.8% ± 4.4 (median ± SEM) across intestinal segments through histological analysis^26^ and fluorescence-activated cell sorting (FACS) using mice expressing fluorescent protein, mKate2, under the control of the enterocyte-specific *Villin-1* promoter (see Supplementary methods and Supplementary Fig. 1). DNA modification state in the villi enterocytes was examined with single nucleotide resolution in the *Lct*–*Mcm6* locus of mice using the bisulfite padlock probe technique^27^. Padlock probes (n = 314) were designed to target non-repetitive genomic sequences on both DNA strands (GRCm38/mm10; Supplementary File 1). Enrichment of targeted bisulfite-converted DNA was done as described^27^ (see Supplementary methods), and sequencing was performed on an Illumina HiSeq 2500. Preprocessed reads were mapped onto a bisulfite-converted mouse genome (GRCm38/mm10) using Bismark^56^. Modification estimates were included only for cytosines with > 30 reads. DNA modification density was interrogated at 7,580 modified cytosines (609 CpG sites) across 148 unique samples, and an additional 19 replicates. All data are available from the NCBI Gene Expression Omnibus (GEO) database under accession number GSE76373.

### Statistical analysis of DNA modification data

All statistical analyses were performed with R statistical software. To investigate whether DNA modifications of isolated villi enterocytes were associated with transcriptional variation in *Lct* in enterocytes across the small intestine, we checked for normality at individual cytosines using a QQ plot, and performed a Pearson’s correlation between DNA modification density and steady-state *Lct* mRNA levels for each individual cytosine, using the 9 segments of the small intestine (n = 53, 5–6 mice per segment). Significance was set at *p* < 0.01 after Bonferroni correction for multiple testing. Clusters of significantly associated modified cytosines contained 3 or more cytosines within 500 bp. *P*-values are expressed as the –log *p*-value of the correlation coefficient, with the sign ( + /−) representing the direction of Pearson’s correlation (SLP). Genomic sites with DNA modification clusters significantly associated with *Lct* transcriptional variation were further investigated. At these sites, significant changes in the average % DNA modification status in enterocytes across the intestine was determined by one-way ANOVA. *Mcm6* mRNA (Fig. 1b) and DNA modifications at negative control regions in *Mcm6 (Mcm6* intron 1; Fig. 2) did not show significant differences between intestinal segments. No significant sex differences were observed as determined by repeated measures (RM)-ANOVA. The sequences were aligned to small intestine (adult 8 weeks) ENCODE tracks for CTCF (ENCSR000CED), H3K4me1 (ENCSR000CCR), H3K4me3 (ENCSR000CCS), H3K27ac (ENCSR000CCQ), and MEL cell line, GATA-1(ENCSR000EUG).

For testing age-associated change in DNA modifications, segment 1, 3 and 7 of P6 infants were compared against corresponding segments in P60 adults (n = 3–6 per segment per age group). We determined which genomic sites showed significant age-associated differences by analyzing change in DNA modification (adults P60 minus infants P6). Diet-associated DNA modification changes were identified by comparing LAC^+^ (n = 34; 6–7 per segment) and lac^−^ (n = 31; 5–7 per segment) P90 mice. A group-wise variance matrix showed that heavily modified CpGs (>90%) lacked deviation and these were removed from analysis in this experiment (Supplementary Table S1). Significant age- and diet-induced DNA modification changes were identified after Bonferroni correction.

### Chromatin immunoprecipitation of CTCF

Chromatin immunoprecipitation (ChIP) was performed to investigate CTCF binding in intestinal segments 1, 3, 5, 7 and 9 of both P6 and P60 mice (n = 3 per group). Tissue homogenization and ChIP were performed using the MAGnify ChIP kit (Life Technologies). Immunoprecipitation was performed overnight, using 3 μl monoclonal CTCF antibody (Pierce G.758.4), and negative control reactions used 1 μg of mouse IgG antibody (Life Technologies). Input controls were also taken for each sample. qPCR was performed with Universal SYBR Green Supermix (Bio-Rad) in triplicate for four *Lct*–*Mcm6* loci and two negative control locations up/downstream of this site (primers listed in Supplementary Table S2).

## Additional Information

**Accession codes:** Bisulfite sequencing data can be access on GSE76373.

**How to cite this article**: Oh, E. *et al*. Transcriptional heterogeneity in the lactase gene within cell-type is linked to the epigenome. *Sci. Rep.* **7**, 41843; doi: 10.1038/srep41843 (2017).

**Publisher's note:** Springer Nature remains neutral with regard to jurisdictional claims in published maps and institutional affiliations.

## Supplementary Material

Supplementary Information

Supplementary File
