# Supplementary material for: Transcriptional heterogeneity in the lactase gene within cell-type is linked to the epigenome
Source: Sci Rep. 2017 Jan 31;7:41843. doi: 10.1038/srep41843 (PMC5282553; doi:10.1038/srep41843)
Supplement: Supplementary Information [file srep41843-s1.pdf]

Transcriptional heterogeneity in the lactase gene within cell-type is linked to the epigenome

Edward Oh[1,4], Richie Jeremian[1,4], Gabriel Oh[1], Daniel Groot[1], Miki Susic[1], KwangHo Lee[2], Kelly Foy[2], Peter W. Laird[2], Arturas Petronis[1,5,*], Viviane Labrie[1,3,5,*].

[1]Krembil Family Epigenetics Laboratory, Centre for Addiction and Mental Health, Toronto, Ontario, Canada

[2]Center for Epigenetics, Van Andel Research Institute, Grand Rapids, MI, USA

[3]Center for Neurodegenerative Science, Van Andel Research Institute, Grand Rapids, MI, USA

[4]equal contribution (first authors)

[5]equal contribution (senior authors)

*Correspondence: Art.Petronis@camh.ca (A.P.) and Viviane.Labrie@vai.org (V.L.)

Keywords: Epigenetics; DNA methylation; DNA modification; Lactase gene; mRNA; Small intestine; Enhancers; Aging; Cell heterogeneity

SUPPLEMENTARY MATERIAL

Supplementary File 1 (Oh_Supp_File_1_PadlockProbes.xlsx)

Supplementary PDF list of content:

Detailed Supplementary Material and Methods

Supplementary References

Supplementary Table 1

Supplementary Table 2

Supplementary Figure 1

Detailed Supplementary Material and Methods

Mouse intestinal samples and milk treatments

All animal procedures were approved by the Institutional Animal Care Committee of the Toronto Centre for Phenogenomics (TCP) and compiled per the requirements of the Canadian Council on Animal Care and Province of Ontario Animals for Research Act. C57BL/6NCrl mice were bred at the TCP. Mice were housed in ventilated polycarbonate cages, and given *ad libitum* sterile food (Harlan 2918X) and water, unless stated otherwise. Adult mice were housed by sex in groups of 2-5 littermates. The vivarium was maintained under temperature (21°C±1°C) and humidity (50-60%), with a 12-hour diurnal cycle (lights on 0700-1900).

Infant mice at postnatal day 6 (P6) and adult mice at postnatal day 60 (P60) were used to investigate the segmental gradient and age-associated epigenetic changes. The small intestine was harvested and dissected into nine equal parts; the duodenum (segment 1-2), jejunum (segment 3-5) and ileum (segment 6-9). Each of these anatomical segments was further dissected into three equal parts resulting in nine dissections of the small intestine (segments 1-9). To investigate the diet/environmental associated epigenetic changes, postnatal day 30 mice were supplied *ad libitum* sterile food with either 2% lactose-containing organic milk (Grand Pré) or 2% lactose-free milk (Natrel) in the place of water for 60 days. Approximately 500 ml of lactose-containing milk (LAC+) was supplemented with 1 tablespoon (or ~12 g) of sucrose (Redpath) to promote a consumption level comparable to that of mice fed lactose-free milk every 1.5~2 days. No sex differences were observed in either DNA modification or *Lct* mRNA expression level. Mouse intestinal samples were processed by researchers who were blind to genotype and experimental conditions.

Enterocyte isolation and DNA extraction

Enterocytes were isolated specifically from the villi of each small intestine segment (approx. 100 mg) using a previously validated method[1,2]. The segments were washed four times in 1 mL citrate buffer (27 mM Na-citrate, 5.6 mM Na_2HPO_4, 96 mM NaCl, 8 mM KH_2PO_4, 1.5 mM KCl, pH 7.4) containing 10 µl of DNase inactivation reagent (Life Technologies) with gentle agitation. Enterocytes were released with the addition of 1 mL Ca^{2+}-chelating buffer (1.5 mM EDTA, 0.5 mM DTT, 10 mM NaH_2PO_4, 154 mM NaCl) at 37°C for 15 min with agitation (700 rpm). The intestinal segments and suspended enterocytes were separated, and the enterocytes were collected by centrifugation at 1000 rpm for 5 mins at 4°C. This was repeated twice using fresh Ca^{2+}-chelating buffer for maximal recovery of villi enterocytes. The purity and specificity of the villi enterocyte isolation was confirmed using histological investigation (Labrie et al., 2016) and fluorescence-activated cell sorting (FACs; see below). DNA was extracted from enterocytes and the small intestinal segment lacking enterocytes using standard phenol-chloroform DNA extraction methods in combination with Phase-Lock tubes (5 Prime) after an overnight treatment in Proteinase K. DNA quantity was measured by NanoDrop ND-1000, and DNA quality was verified on a 1% agarose gel.

Fine-mapping DNA modifications in enterocytes

DNA modification state was examined with single nucleotide resolution in the *Lct* and *Mcm6* loci of mice using the bisulfite padlock probe technique[1,3]. All investigations of DNA modifications were performed on isolated villi enterocytes. Whole genome amplified DNA (WGA; Sigma), a genome devoid of DNA modifications, was also bisulfite-converted and used to verify the efficiency of the bisulfite conversion reactions; we found ~98% conversion in samples. 314 padlock were targeted to unique (non-repetitive) genomic sequences (GRCm38/mm10). Probes

covered 90kb of the region of interest on both forward and reverse DNA strands. Target DNA regions were designed for bisulfite converted DNA using ppDesigner software. CpG sites within probes were modified to capture both modified and unmodified cytosines. Probe sequences are found in Supplementary File 1. Using a custom pipeline based on the Bismark tool[4], DNA modification density was interrogated at every cytosine covered by padlock probes. Reads were first trimmed and residual adapter sequences removed using Trimmomatic-0.32. To remove phiX sequencing control DNA, reads were aligned to the phiX reference genome (NC_001422.1) using Bismark (560 bp from the beginning of the phiX genome was appended to the end to accommodate the circular nature of the genome for read alignment). Reads that did not align to phiX were aligned to the target reference genome (GRCm38/mm10). Percent modification was estimated as the fraction of spanning reads that retained the reference "C", and were not converted to "T" from the bisulfite treatment. Modification estimates were included only in those cytosines which received 30 or more reads. DNA modification density was interrogated at 7,580 cytosines (609 CpG sites inclusive) across 148 unique samples, and additional 19 replicates. DNA modification sites were analyzed for outliers and sparsity issues (coverage in fewer than half of the samples), but none were found, and no sample-wise outliers were found by 1.5IQR limits. Biological variation exceeded technical noise; the mean deviation of DNA modifications between randomly paired samples (average standard deviation, 0.0414±0.0114) was higher than between were same sample replicates (average standard deviation, 0.0232±0.0069). All data are available from the NCBI Gene Expression Omnibus (GEO) database under accession number GSE76373.

Flow cytometry analysis of isolated enterocytes

Purity of isolated enterocytes using the chelating method was determined by flow cytometry analysis, using transgenic mice that express an mKate2 fluorophore in enterocytes only. The construct for the *Villin-mKate2* transgenic line was generated using the intestine-specific

mouse *Villin-1* promoter excised from the pBSKSVillinMESSV40polyA vector (gift from Dr. Louvard) and a *mKate2* gene purchased from the Evrogen. The construct was then injected into B6D2F1 hybrid mouse male pronucleus at the USC transgenic core facility. Isolated enterocyte pellets were resuspended in 5ml Accutase (STEMCELL Technologies) and incubated at 37°C for 20 mins. Accutase treated cells were then centrifuged at 300xg for 5 minutes and the resulting cell pellets were resuspended in 500 µl PBS for flow sorting. Single-cell suspensions were stained with DAPI (4′,6′-Diamidino-2-phenylindole) and alive enterocytes were used for subsequent sorting. Purity of enterocytes following our chelating protocol was determine by comparing the same intestinal segments from a *Villin-mKate2* mouse to those of a mouse littermate lacking *mKate2* (Supplementary Fig. 2). The Van Andel Research Institute Flow Cytometry Facility examined cells on a CytoFlex S Flow Cytometer (Beckman Coulter Inc.) and data were analyzed using FlowJo (FlowJo LLC.).

Chromatin immunoprecipitation of CTCF

Chromatin immunoprecipitation (ChIP) was performed to investigate CTCF binding in intestinal segments 1, 3, 5, 7 and 9 of both P6 and P60 mice (n=3 per group), as well as segment 7 of LAC^{+} and lac^{-} milk-fed mice (n=3 per group). Tissue homogenization and ChIP were performed using the MAGnify ChIP kit (Life Technologies) per the manufacturer’s instructions. Immunoprecipitation was performed overnight, using 3 µl monoclonal CTCF antibody (Pierce G.758.4) per ChIP reaction, and 1 µg of mouse IgG antibody (Life Technologies) for negative control reactions. A non-immunoprecipitated input control was also prepared for each sample. qPCR was performed in triplicate on all samples for five loci: *Mcm6* exon 13/intron 13 and exon 7, as well as *Lct* exon 1, intron 2 and intron 8. This was done using the SsoAdvanced Universal SYBR Green Supermix (Bio-Rad) using the Applied Biosystems ViiA 7 real-time PCR system. The reaction was carried out per the following conditions in a 20 µl total reaction volume: 0.9X

Universal SYBR Green Supermix, 10 µl recovered sample, 0.25 µM forward and reverse primer (primer sequences detailed in supplemental table 2); 98°C for 3 min and 50 cycles of (98°C for 15 sec, 55°C for 1 min). Percent input method with background normalization was calculated for further analysis.

Supplementary References

1 Labrie, V. *et al.* Lactase non-persistence is directed by DNA variation-dependent epigenetic aging. *Nat Struct Mol Biol* (2016).

2 Weiser, M. M. Intestinal epithelial cell surface membrane glycoprotein synthesis. I. An indicator of cellular differentiation. *J Biol Chem* **248**, 2536-2541 (1973).

3 Diep, D. *et al.* Library-free methylation sequencing with bisulfite padlock probes. *Nat Methods* **9**, 270-272, doi:10.1038/nmeth.1871 (2012).

4 Krueger, F. & Andrews, S. R. Bismark: a flexible aligner and methylation caller for Bisulfite-Seq applications. *Bioinformatics* **27**, 1571-1572, doi:10.1093/bioinformatics/btr167 (2011).

Supplementary Tables

Supplementary Table S1: Diet-associated DNA modification between LAC+ (n=39; 7-9 per segment) and lac- (n=35; 5-9 per segment) investigated by Pearson's and variance matrices within each condition by modification status of individual CpGs.

**Average R^2 (Pearson)**	**All CpGs**	**>90% meth**	**90-20% meth**	**<90% meth**	**<20%meth**
lactose-free milk vs itself	0.9863	0.8703	0.9497	0.9824	0.8224
Normal milk vs itself	0.9899	0.8951	0.9563	0.9871	0.9130
lactose-free vs Normal milk	0.9893	0.8652	0.9595	0.9863	0.8502
Change in avg R^2	*0.003586*	*0.024848*	*0.006649*	*0.004673*	*0.090554*
**Average Variance**	**All CpGs**	**>90% meth**	**90-20% meth**	**<90% meth**	**<20%meth**
lactose-free milk vs itself	0.0705	0.0003	0.0313	0.0927	0.0016
Normal milk vs itself	0.0714	0.0004	0.0265	0.0925	0.0023
lactose-free vs Normal milk	0.0705	0.0003	0.0288	0.0919	0.0019
Change in avg variance	*0.000837*	*0.000055*	*-0.004813*	*-0.000207*	*0.000708*

Supplementary Table S2: CTCF ChIP-qPCR primer locations

**Primer**	**Locus**	**Sequence**	**Location**
x1-FWD	*Lct* exon 1	TCTCAACAAGGTCCCCAAAG	chr1:128327796-128327815
x1-REV	*Lct* exon 1	TGCAGTGCTACCGACAACTC	chr1:128327950-128327969
i2-FWD	*Lct* intron 2	TCCTGCACAGAAGTGAGCTG	chr1:128313174-128313193
i2-REV	*Lct* intron 2	CCATAGCCAGCATACCCATC	chr1:128313309-128313328
i8-FWD	*Lct* intron 8	ATCCTTTCCGTGTCATCCAG	chr1:128299877-128299896
i8-REV	*Lct* intron 8	AGCCTTGATCTCCATGATGC	chr1:128300007-128300026
Mcm6-FWD	*Mcm6* intron/exon 13	ACTCATGCATTCCTGTTCCC	chr1:128338074-128338093
Mcm6-REV	*Mcm6* intron/exon 13	AGTACAAACGTCTCCGCCAG	chr1:128338224-128338243
Mcm6-X7-FWD	*Mcm6* exon 7	GAGGCTCGTGCAGAGATTGT	chr1:128348385-128348404
Mcm6-X7-REV	*Mcm6* exon 7	CCACTTCAGTTTGGAGGGAA	chr1:128348509-128348528
Negative control A: noCTCF-up-F		GTCATAGTTCCCTGGGCAAA	chr1:128490456-128490475
Negative control A: noCTCF-up-R		CAACAGGATCCAGGGTCAGT	chr1:128490568-128490587
Negative control B: noCTCF-down-F		CCCGAAGGAAAACAGGTGTA	chr1:127891693-127891712
Negative control B: noCTCF-down-R		GTTCAGAGCAAGCCCAAGAC	chr1:127891856-127891875

Supplementary Figures

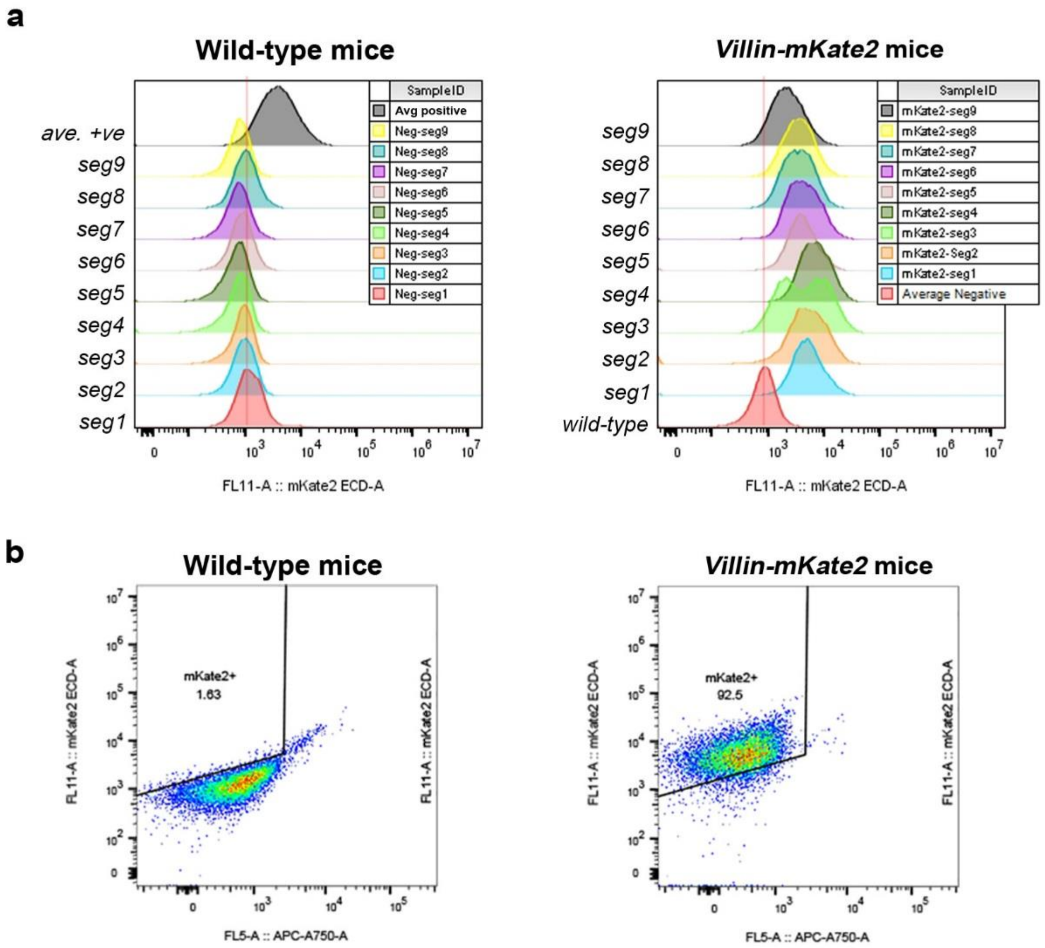

Supplementary Fig. 1: Flow cytometry analysis of purity of enterocytes that were isolated using the chelating method. Enterocytes were isolated from *Villin*-mKate2 expressing mice and wild-type littermates by intestinal segments. **a)** Histogram depicting isolated enterocytes from wild-type mice (left) and mice expressing mKate2 under the control of the enterocyte-specific *Villin* promoter (right). Each of the nine intestinal segments were examined for mKate2 fluorescence (x-axis) in wild-type and *Villin*-mKate2 mice. Average positive and average negative refers to the average mKate2 signal across all intestinal segments of *Villin*-mKate2 and wild-type mice, respectively. **b)** Representative dot plot of isolated enterocytes. Using the chelating method, we were able to achieve ~84.8% purity in the intestinal segments. Y-axis: mKate2 fluorescence channel.
